# The Students’ Flow Experience With the Continuous Intention of Using Online English Platforms

**DOI:** 10.3389/fpsyg.2021.807084

**Published:** 2022-02-08

**Authors:** Hong Zhao, Asif Khan

**Affiliations:** ^1^International College of Cultural Education, Northeast Agricultural University, Harbin, China; ^2^Department of Marketing and Distribution Management, College of Management, National Kaohsiung University of Science and Technology, Kaohsiung, Taiwan

**Keywords:** online English learning, flow experience, antecedents of flow, flow theory, expectation confirmation model, ECM, continuous intention

## Abstract

Built on the integrated theoretical framework of antecedents of flow and expectation confirmation model (ECM), this research investigates the way flow experience drives the online students’ intention to engage in online English teaching platforms. This study focused on the online students engaged in online English learning platforms in Taiwan. A total of 500 online students were selected. An online survey was conducted with the help of a marketing research agency located in Taiwan. According to the results, the online students’ flow experience was found to be in a significant relationship with continuous intention. The antecedents, including perceived enjoyment, challenge, and situational involvement, were found to be in a positive relationship with flow experience; however, confirmation and perceived vividness did not have significant effects on the flow. Furthermore, flow and confirmation were found to be in a significant relationship with perceived usefulness and satisfaction. Moreover, perceived usefulness was found to be in a significant relationship with satisfaction and continuous intention. Lastly, satisfaction was found to be in a significant relationship with continuous intention. Finally, the questions proposed in this research with their empirical findings offer profound understanding for establishing a well-devised online English learning platform that can motivate online learning. These results and managerial implications for online English language platforms are innovative and significant in practice.

## Introduction

Nowadays, online learning platforms offer an educational atmosphere to learners and individuals concerned with enhancing their knowledge about the current advancements in certain disciplines. These online platforms are utilized by a huge number of individuals simultaneously, not including any time, or location limitations. An evolving question rising from offering learning opportunities to various types of learners is the model of an online platform that can explain the activities of its users to adjust its content corresponding to user conduct and requirements. Preferably, an online learning platform will maintain a track of the activities that its users produce while using it and present recommendations to the learner to attain the education results in a well-programmed approach. Therefore, learner modeling is particularly vital to enhance the learning power of an online learning platform (Semerci and Goularas, 2021). To recognize the online English learning behavior of students, research scholars have frequently opted the analysis that is generally applied to the evaluation of conduct in traditional media; however, they have additionally studied different characteristics that are most relevant to online environments as well. The most relevant of these concepts is the theory of flow, which is crucial for its impact on describing virtual experiences (Novak et al., 2000). The concept of flow was introduced by Csikszentmihalyi and Csikzentmihaly (1990), which he described as the universal experience that individuals feel whenever they engage in an activity with complete involvement. The individuals in a state of flow are immersed in an activity, the concentration of their consciousness is reduced, and they believe to be in control of their situations. Research has described flow encounters in various pursuits, including reading, dancing, rock climbing, chess, etc. Provided the reduced figures of returning users, studies that examine the crucial issues that affect continuance use intent are vital to individuals who formulate marketing tactics for online platforms (Lee and Kim, 2020). The sustainable use of online platforms in business processes is critical to generating commercial value. Consequently, sustainability concerns can be employed in creating considerations influencing the continuous use of online platforms (Park et al., 2021). This research paper tends to find the correlation between flow experience and the continuous intention of students using online English learning platforms.

According to the literature on the online flow (Esteban-Millat et al., 2014), enabling optimum online navigation is a distinctive trait of flow that can extend students’ online sessions (Hsu et al., 2012b) and improve the educational performance (Kiili, 2005; Pearce, 2005). The online English learning environment is a field of particular significance, chosen as a consequence of its increasing significance in the delivery of services created by educational organizations. Throughout navigation across an online English learning atmosphere, learners might encounter flow experiences (Pearce, 2005; Choi et al., 2007; Joo et al., 2012). This is deemed necessary to that extent as it establishes an optimum experience through which users understand that the challenges encountered by them are in equilibrium with their skills. Perceived challenges can be described as the prospects for engagement with which a learner is introduced in the platform. During flow, students likewise participate and concentrate on the activity being executed and enjoy the activity, losing consciousness of any additional unrelated environmental stimulus (Esteban-Millat et al., 2014). Furthermore, the perceived vividness related to the design of the online platform and the level of situational involvement by the students is deemed to be important factors in generating flow experiences of students using online English learning platforms (Chang, 2017; Barhorst et al., 2021). Based on the existing studies, this research utilizes the antecedents of measuring consumer flow as perceived enjoyment (Atombo et al., 2017), perceived vividness (Barhorst et al., 2021), challenge (Larche and Dixon, 2020), situational involvement (Chang, 2017), and confirmation (Cheng, 2014).

Furthermore, the expectation confirmation model (ECM) recommends the two key factors of perceived usefulness (PU) (post-adoption belief) and confirmation (initial-adoption belief) that are mutually associated with satisfaction and continuous intention. Furthermore, confirmation additionally signifies a relation to PU. Confirmation related to the performance indicates a confirmation concerning perceived and expected performance of utilizing online platforms (Halilovic and Cicic, 2013). Online students recognize it as an essential motivation for learning purposes on online English learning platforms. PU can be described as the degree to which a specific item or a device is exceedingly useful for people to complete activities. Online students would anticipate increasing the confirmation for a PU of online learning platforms (Elwalda et al., 2016). Moreover, online students’ satisfaction is an essential factor in an inclination to engage in online English learning platforms. Furthermore, flow experience and satisfaction are characterized as emotive and rational viewpoints of students using online platforms. Hence, there is a significant relationship between these variables (Rose et al., 2012; Foroudi et al., 2018; Sharma and Sharma, 2019; Wu et al., 2020). Additionally, learners’ degree of flow produced by the online English learning platform tools substantially impacts their PU that explains their usage intent of the online platform, and it can significantly impact their usage intent related to the online learning platform. Accordingly, a research study concludes that nurses’ confirmation of prospects to the online learning platform had a substantial outcome on their flow experience produced by the online platform, and nurses’ flow experience produced by the online learning platform had substantial impacts on their PU, continuance intention, and satisfaction of the online platform (Cheng, 2014). Consequently, this research analyzes the impact of flow on PU and satisfaction and explores the relationship of confirmation and PU with satisfaction and continuous action.

Precisely, these study constructs for these issues are centered on specific theories. First, the expectation-confirmation model (ECM) has been extensively implemented to analyze the features that impact online platforms utilization (Bhattacherjee, 2001). Flow theory was frequently utilized to secure users’ emotive states concerning the equilibrium of task skills and challenges for an online learning experience (Bilgihan et al., 2014; Wu et al., 2020). This research study covers the following gaps. First, this research paper examines the effect of students’ flow experience on the continuous intention of using online English learning platforms. Second, this study explores the impact of perceived enjoyment, perceived vividness, challenge, situational involvement, and confirmation as antecedents of flow experience. Third, this study assesses the influence of flow on PU and satisfaction. Fourth, this research adopts ECM and analyzes the relationships of confirmation and PU with satisfaction and continuous intention. Finally, this research analyzes the impact of online students’ satisfaction on online English learning platforms’ continuous intention.

## Theoretical Background and Hypothesis Development

### Flow Theory

Flow can be defined as the “optimal experience” that individuals feel whenever they engage with complete involvement. Students in a state of flow utilize their psychological or physical capabilities to engage in behaviors with high concentration, complete engagement, and satisfaction. Additionally, this state can be described by the total concentration in the activity, having no feeling regarding the time and space (Michailidis et al., 2018). Built on a person’s skill and action’s level of challenge, Csikszentmihalyi and Csikzentmihaly (1990) split the previous model of flow into three types of conditions, including flow, boredom, and anxiety. Soon After, this framework was extended into eight conditions, consisting of flow, arousal, relaxation, control, boredom, worry, apathy, and anxiety, individually relating to a mixture of skills and challenge levels (Csikszentmihalyi, 1997). Moreover, flow is also linked to several aspects, for instance, an individual’s underlying perception and motivation. Chan and Ahern (1999) indicated that the formation of action related to challenges, feedback, concentration, control, and goal has significant effects on motivation that may modify if some of its aspects were altered. On the other hand, Lu and Lien (2020) studied learners’ experiences with education in a computer game-centered educational setting and discovered that a greater view of education as “playing” is one of the crucial aspects that can cause flow, improving knowledge effectiveness. Furthermore, Csikszentmihalyi and Csikzentmihaly (1990) defined eight factors that influence flow, for instance, clear aims and instantaneous feedback, a balance between the skill and challenge levels, integration of activity and consciousness, absorbed attentiveness, a feeling of control, time misrepresentation, a loss of self-awareness, and being self-gratifying. In reflecting these aspects for the appearance of a state of flow, Csikszentmihalyi and Csikzentmihaly (1990) and Arzate Cruz and Ramirez Uresti (2018) claimed that the state of flow might be brought even after merely a few of the considerations are fulfilled. In addition, several researchers have discovered that the balance of challenge skills specifically involves the manner students attain flow experiences (Shernoff et al., 2014; Wang et al., 2014). Flow is a psychological condition, which can stimulate the internal impulse to produce a high level of concentration and involvement. Furthermore, it can also enhance an individual’s mind or body to its thresholds to engage in tough and valued activities, regularly boosting excellent work productivity. Several studies have demonstrated the significance of flow in improving learners’ attitudes, performance, and motivation (Schweinle et al., 2008; Heutte et al., 2016; Tavares and Freire, 2016). Consequently, several investigations have investigated learners’ conditions while being in a state of flow and its impacts on education (Hamari et al., 2016; Wu et al., 2021).

### The Expectation Confirmation Model

Expectation confirmation model introduced by Bhattacherjee (2001) incorporates expectation confirmation theory (ECT) and PU to study the intent of continued usage in the framework of information systems. Bhattacherjee (2001) claimed that a student’s continuous intent to utilize an information system is usually related to the reasoning behind the student’s reuse intention in ECT. The ECM has been commonly implemented to inspect online platforms’ usage for managing design structures (Lu et al., 2019). In particular, the ECM recommends the two key influencers of confirmation and PU that are mutually connected to the online students’ satisfaction and continuous intent to utilize the online English learning platform. Additionally, confirmation likewise suggests a connection to PU. Confirmation describes an association between the anticipated and perceived performance of utilizing online English learning platforms, such as course description and navigation (Halilovic and Cicic, 2013). Particularly, online students recognize it as a significant inspiration for English learning on online platforms. On the other hand, PU is related to the usefulness of an object in fulfilling a certain task. Online students might increase their performance levels to ensure the usefulness of online English learning platforms (Elwalda et al., 2016). Online students’ satisfaction is also deemed as an essential factor to engage in online English learning activities (Wu et al., 2020). Fundamentally, personal characteristics specify a person’s mental state and describe the manner a flow state is achieved. Numerous scholars have claimed challenge as a personal characteristic associated with flow experience (Kamis et al., 2008; Hausman and Siekpe, 2009). In an online setting, a task challenge suggests a student’s emotional experience for a reaction to activity. Consequently, task challenges describe concerns of a person’s emotive state (Wu et al., 2020).

### Flow and Continuous Intention

Csikszentmihalyi describes flow as a feeling that individuals experience when they are completely involved in certain behavior. Flow can be achieved while being engaged in several daily activities, for instance, sports, watching movies, or reading (Pelet et al., 2017). It is additionally believed that individuals that feel pleasure while being in a state of flow may grow an inclination to repeat the conduct during an action. In a state of flow, time might appear to hold still when individuals are involved in a certain activity (Park et al., 2021). Irrespective of the particular age group, the fundamental explanation that students use online English learning platforms is because online platforms expand students’ satisfaction by offering improved value to the users, thus causing continuous usage of online English learning platforms (Kwak et al., 2014). The intent to utilize a specific service varies on the user’s evaluation of the facility that, in line, influences the determination to continue utilizing the service. To generate workable value for the future and to produce profits, policies that improve the reuse intent or satisfaction of users that utilize a certain facility are required, and the examination must be constantly conducted (Campbell et al., 2014; Hwang and Cho, 2018; Park et al., 2021). Based on the mentioned literature, the following hypothesis can be postulated.

**Hypothesis 1:** Online students’ flow has a significant impact on continuous intention to use.

### Antecedents of Flow Experience

This research study targets the essential antecedents of flow, including confirmation, perceived enjoyment, perceived vividness, perceived challenge, and situational involvement with the flow theory.

#### Confirmation and Flow

Confirmation linked to the performance implies a confirmation regarding perceived and anticipated performance of employing online platforms (Halilovic and Cicic, 2013). Essentially, the ECM is demonstrated to have great analytical validity for the information technology-related continuance intention, and it is considered as a reliable framework principally from the extrinsic motivational point of view (Hong et al., 2006; Thong et al., 2006). Nevertheless, the ECM delivers slight support in taking the confirmation of students’ intrinsic motivation in the utilization of online learning platforms that can be a crucial factor influencing the students’ continuance intention; therefore, including the intrinsic motivator into the ECM might deliver an improved description of the online learning platform’s continuance intention (Thong et al., 2006; Sørebø et al., 2009). Once students utilize the collaborative systems, for instance, video conferencing, messenger, or discussion rooms, etc., offered by online English learning platforms to connect with other individuals, they can be in a state of flow as these platforms may make them feel completely engaged in their activities (Lee, 2010). The users’ confirmation of prospects concerning the online learning platform has a significant influence on their degree of intrinsic motivation regarding the usage of online learning platforms; furthermore, users’ degrees of intrinsic motivation related to online learning platforms usage can significantly impact their degrees of satisfaction with online learning platforms usage and continuous intention of utilizing the online learning platform (Sørebø et al., 2009). Later, once students are completely engaged in their online English learning activity, and experience the enjoyments of collaborations with the online learning platform, they may realize that such an online learning platform is beneficial (Saadé and Bahli, 2005; Cheng, 2014). Based on the provided literature, the following hypothesis can be postulated.

**Hypothesis 2:** Online students’ confirmation has a significant impact on their flow experience.

#### Perceived Enjoyment and Flow

Perceived enjoyment is described as “the degree of perceived enjoyment experienced while using a particular technology to perform an activity, apart from some performance effects stemming from technology usage” (Venkatesh, 2000). Individuals are fundamentally motivated to participate in action once they enjoy utilizing technology to perform a certain behavior. Likewise, advanced online English learning platforms utilize a great amount of technology-linked elements that can create excitement and enjoyment for students throughout usage. Particularly, some students’ use of innovative online learning platforms might be fundamentally motivated to involve in pleasurable online learning activities to make their experience enjoyable. Another study additionally demonstrated that, while, in a state of flow, individuals feel a perception of control across their activities (Li and Browne, 2006). Consequently, in an enjoyment temperament, if a student has a great intent to execute an action, the student might believe to possess the required means and abilities to engage in those behaviors (Atombo et al., 2017). Enjoyment and concentration have been applied to predict individual intention and behaviors (Lee and Chen, 2010) established on the fact that users are further prone to be motivated to continuously engage in any behavior that is deemed as enjoyable as compared to any similar behavior that is not perceived as enjoyable. Additionally, in the attainment of a certain goal, an individual should focus on the activity and ignore everything else (Nakamura and Csikszentmihalyi, 2014; Atombo et al., 2017). Hence, we can postulate the following hypothesis.

**Hypothesis 3:** Online students’ perceived enjoyment has a significant impact on flow.

#### Perceived Vividness and Flow

Vividness is described as “the capability of a technology to create an astoundingly powerful facilitated environment.” It implies the method of merging the sensual understanding of real items that can be viewed with the non-sensual imaginary items generated in a person’s thinking to build a clear-cut vision of an encounter (Barhorst et al., 2021). Flavián et al. (2017) theorize that vivid knowledge can occur in several types, comprising audio, images, and visual substance that arouses the practical and physical characteristics of an acquisition. In an online scenario, vividness is frequently linked along with the visual appeal of the online learning platform (Flavián et al., 2017). An extra vivid presentation is additionally expected to impact a student’s intellectual processing because of its additional fascinating appeal, causing an improved assessment of the required data than what dull data would generate. Appropriately, the vividness of the knowledge can enhance the view of material quality *via* growing the number of sensory elements that, in turn, might improve cognitive handling. Vividness, as compared to interactivity, facilitates online students to mentally envision the forthcoming experiences (Phillips et al., 1995; Barhorst et al., 2021). As stated previously, users enjoy an online platform (Rauschnabel et al., 2017) and sense a feeling of uniqueness and magnificence of an event because of the vividness of the event (McLean and Wilson, 2019). A study, furthermore, discovered the significant role of perceived vividness in online augmented reality of AR technologies by studying flow in an AR and a conventional shopping perspective (Barhorst et al., 2021). Based on the mentioned literature, the following hypothesis can be postulated.

**Hypothesis 4:** Online students’ perceived vividness is significantly related to flow.

#### Challenge and Flow

Various kinds of research have demonstrated that, when challenges and skills are almost equivalent, the state of flow is attained. Even for novices with fewer skills, if they are engaged in an uncomplicated activity, they must experience flow because their negligible skills are paired with the negligible challenges of the activity. Furthermore, if the flow is a greatly motivating condition, then even comparatively beginner performers must be encouraged to continue performing precisely from the beginning to a novel English learning experience. Practical support for the idea that flow contained by online learning platforms is generated by the equilibrium of skills and challenges that come from different kinds of research that influences the extent to which an activity is deemed as challenging in an online learning platform by raising or lowering the speed at which students must engage (Keller and Bless, 2008; Keller et al., 2011; Jin, 2012; Kennedy et al., 2014; Harmat et al., 2015; Baumann et al., 2016; Tozman et al., 2017; Larche and Dixon, 2020; Wu et al., 2021). Specifically, Shernoff et al. (2014) emphasized that commitment is possibly improved after a person recognizes the task challenge and his or her abilities as a high level and stable. Based on the mentioned literature, the following hypothesis can be postulated.

**Hypothesis 5:** Online students’ challenges have a significant impact on flow.

#### Situational Involvement and Flow

Situational involvement signifies short-term emotions of enhanced involvement that is associated with a specific situation (Rothschild and Houston, 1980). Social judgment theory was initially established in the middle of the 20th century to examine relations between behavior and ego involvement (Sherif and Cantril, 1947). Involvement was implemented by scholars in the middle of the 1980s to assess relations with individuals’ relaxation alternatives (Havitz and Dimanche, 1997). Involvement is described by Havitz and Dimanche (1997) as a non-observable condition encouragement, stimulation, or curiosity regarding an entertaining activity, induced by a specific motivation or condition, and which has motivational features. Involvement, at its fundamental point, expresses an individual’s curiosity in a specific action, service, or perspective. It is usually theorized as containing elements of fun, excitement, or pleasure (McQuarrie and Munson, 1987). Situational involvement, as stated previously, is perspective dependent, and, hence, the significance that an individual puts on an activity will vary among different circumstances (Havitz and Mannell, 2005). Although co-involvement might be an influential element influencing the enjoyment an individual attains from contributing to action, no investigation has studied situational involvement and co-participants to explore the interactions among them. However, flow experiences and situational involvement have been reviewed in association with each other. The research study of Havitz and Mannell (2005) described that, if excessive degrees of involvement were specified, higher degrees of flow were also discovered. Situational involvement was observed to impact or mediate relationships with some direct psychological constructs, for instance, enjoyment impacting subsequent leisure conduct (Decloe et al., 2009). Moreover, Lee et al. (2011) indicated that involvement is in a significant relationship with flow experiences generated during complete immersion in activities. Likewise, Decloe et al. (2009) discovered a higher degree of situational involvement in the course of flow-resembling occurrences. Furthermore; Kang et al. (2009) additionally delivered a comparable outcome, suggesting that a high degree of involvement in behaviors generates additional emotional advantages and flow. In summation, previous studies have discovered significant associations between flow and situational involvement (Havitz and Mannell, 2005; Naylor, 2006; Chang, 2017). Hence, the following hypothesis can be postulated.

**Hypothesis 6:** Online students’ situational involvement has a significant impact on the flow.

### Flow and Perceived Usefulness

To match the theory of flow into the association’s basic technology acceptance model, it is recommended that the degree of intellectual dissonance related to the execution of technology tasks is lowered when individuals are in a state of experience flow because they consider that giving time on a specific activity would be useful (Agarwal and Karahanna, 2000). According to self-perception theory of Bem (1972), people manage to justify their activities and attempt to lower the cognitive dissonance, comprising conflicting attitudes, beliefs, or behaviors. While, in the cognitive absorption state, people experience pleasure and satisfaction when they interact with technological activities if they are in a state of cognitive absorption. Furthermore, their conflicts are diminished after being in a pleasant and enjoyable state (Mahfouz et al., 2020; Purwanto and Ismail, 2020; Hyun et al., 2021; Khan and Khan, 2021). Consistent with the concepts stated above, it is assumed that, once students are fundamentally motivated, they would acknowledge online English learning as useful. Hence, the following hypothesis is postulated.

**Hypothesis 7:** Online students’ flow experience will have a positive impact on PU.

### Flow and Satisfaction

Flow experience can be connected to satisfaction if online students are engaged in a specific enjoyable activity related to online learning platforms that would provide a high level of satisfaction (Ali, 2016). In other words, online students may be inclined to lessen the difficulty of the evaluation procedure in order to build a constructive mindset for utilizing online learning platforms. Numerous pieces of research have demonstrated a significant relationship between flow and satisfaction in employing online platforms (Hsu et al., 2012a; Panigrahi et al., 2018; Wu et al., 2020). Research suggested a research model with three phases of impact centered on the Stimulus-Organism-Response theory to investigate the conduct of online users. According to the findings of this study, a positive significant relationship was discovered between flow and satisfaction (Hsu et al., 2014). Additional research created a research framework to assess online buying intent in the hotel business by examining a correlation arrangement with the drivers of website value *via* the mediation flow and satisfaction. The flow state was uncovered as an essential antecedent of satisfaction (Ali, 2016). There is a likewise correlation structure for suggesting enjoyment, which is a kind of flow experience and logical reactions as two mediators to the level of satisfaction attained by the technological and personal driving forces (Rose et al., 2012). Online students’ flow is deemed to have a significant impact on satisfaction (Wu et al., 2020). Hence, the following hypothesis can be postulated.

**Hypothesis 8:** Online students’ flow state is significantly related to satisfaction.

### Confirmation and Perceived Usefulness

The performance confirmation of online learning platforms is described as the initial adoption belief that specifies a dynamic influence to stimulate a student’s PU also referred to as the post-adoption belief related to employing the online platform (Wu et al., 2020). Bhattacherjee (2001) additionally claimed that online learning platform confirmation can perform a crucial role in deciding PU for investigating the continuous usage of an online learning platform. Several findings have applied the ECM as a theoretical source to analyze the usage of online learning platforms, discovering that online platform performance confirmation significantly impacts PU (Kang et al., 2009; Tsao, 2013; Hsu and Lin, 2015; Lu et al., 2019). Particularly, Lu et al. (2019) outlined a research model to study the association of consumer loyalty related to cell phone advertising on the ECM. According to the findings of their study, a positive significant impact was found between the performance confirmation of the online platform and PU. Based on the mentioned literature, the following hypothesis can be postulated.

**Hypothesis 9:** Online students’ confirmation has a significant impact on PU.

### Confirmation and Satisfaction

The ECM suggests a significant relationship of performance confirmation on satisfaction (Oliver, 1980). Therefore, a connection between the online English learning platform and students’ satisfaction can be characterized as established on the theoretical foundation. The ECM recommends a relationship between the performance confirmation of online learning platforms and students’ satisfaction for analyzing continuous platform usage intent (Bhattacherjee, 2001). Precisely, numerous studies have employed the ECM for analyzing this relationship for different online frameworks, containing social commerce, impulse buying, and mobile commerce (Li and Ku, 2018; Lu et al., 2019). In particular, some research studies found a significant positive association between confirmation and satisfaction (Hsu and Lin, 2015; Wu et al., 2020). Hence, the following hypothesis can be proposed.

**Hypothesis 10:** Online students’ performance confirmation has a significant relationship with satisfaction.

### Perceived Usefulness and Satisfaction

Perceived usefulness is described as a post-adoption belief in information technology usage and suggests an essential linkage to satisfaction (Wu et al., 2020). Built on the ECM, students’ PU of the online learning platform has a significant impact on their satisfaction. This association is validated by the adaptation level theory that recommends that students observe motivations only if they are related to an adapted level. Previous marketing research has uncovered that the greater the students’ beliefs, the greater will be their levels of satisfaction (Oliver and DeSarbo, 1988). For online learning activities, students are further expected to create an optimistic attitude for their conduct on online platforms, such as satisfaction, because online learning platforms are deemed as useful for their collaboration to explore and locate information related to the offered services (Luo and Chea, 2018; Panigrahi et al., 2018; Wu et al., 2020). Additionally, the study examined the motivational forces to influence buying intention for paid cell phone applications by employing the ECM in the research model to describe PU as an antecedent of satisfaction. The outcomes described a significant relationship between PU and satisfaction (Hsu and Lin, 2015). Thus, the following hypothesis can be postulated.

**Hypothesis 11:** Online students’ PU has a significant relationship with satisfaction.

### Perceived Usefulness and Continuous Intention

Expectation confirmation theory is employed as a standard model to examine the hypothesized relationships in an online platform’s context. Bhattacherjee used ECT to examine the relationship between satisfaction and continuous intention. According to the outcomes of his study, it was reasonable to employ ECT in the online platform’s context. In addition, post-adoption behavior in online services showed the appropriateness of the study (Lin et al., 2005). Furthermore, the literature related to the adoption of information technology discovered PU as the most crucial factor of the acceptance intentions of users (Wu et al., 2020). Consequently, the ECM postulates users’ PU of online learning platforms has a significant impact on their continuous online platform usage intention (Bhattacherjee, 2001). Based on the mentioned literature, the following hypothesis can be postulated.

**Hypothesis 12:** Online students’ PU has a significant relationship with satisfaction.

### Satisfaction and Continuous Intention

The ECM suggests that a person’s intent to maintain online platform use is reliant on three variables, including the degree of satisfaction, confirmation, and post-adoption beliefs, in the shape of PU. Online learners’ satisfaction has been discovered to have a significant impact on their continuous intention of using online learning platforms. Findings in marketing have found that the main cause for users’ choice to repeat their behaviors is based on their levels of satisfaction (Bearden and Teel, 1983; Oliver, 1993; Szymanski and Henard, 2001; Lee, 2010). Owing to the similarity between the online users’ satisfaction and continued usage, the following hypothesis can be postulated.

**Hypothesis 13:** Online students’ satisfaction has a significant impact on continuous intention.

**FIGURE 1:**
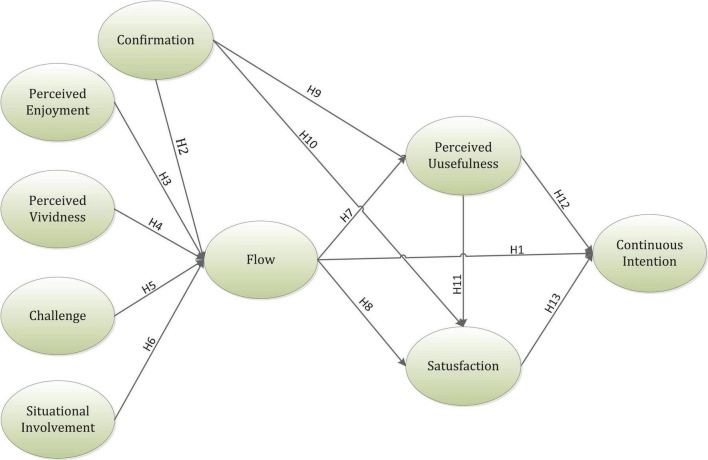
Theoretical framework of the research.

Figure 1 indicates the research framework for this article.

## Methodology

### Sample and Procedure

This study focused on the online students engaged in online English learning platforms in Taiwan. A total of 500 online students were selected. An online survey was conducted with the help of a marketing research agency located in Taiwan. The convenience sampling technique was used by the marketing agency to collect the data from the online students. A close-ended questionnaire was circulated *via* emails to the students, and valid responses were collected with a response rate of 92.48%, which is usually considered to be an optimum response rate (Comrey and Lee, 1992; MacCallum et al., 1999). Rather than asking respondents simply whether they agree or accept an opinion statement, Likert scale items asked how strongly they agree or disagree with it, usually on a 7-point scale from 1 (= strongly disagree) to 7 (= strongly agree), with 4 being a neutral feeling or category. The flow experience was measured by the items adopted from the research study of Xu et al. (2021). The items to measure confirmation were taken from the research of Cheng (2014). Perceived enjoyment and perceived vividness measurement items were modified and adopted from the study of Barhorst et al. (2021). Furthermore, the challenge construct was measured using the items from the study of Fang et al. (2013). Whereas the items for situational involvement were taken from Havitz and Mannell (2005) research study. Moreover, the items to measure PU, satisfaction, and continuous intention were adopted from the study of Tawafak et al. (2018). Before beginning the formal research survey, a pilot test was performed on fifty randomly selected sample representative companies. The questionnaire items were analyzed and authenticated with the support of this test. The hypothesis of this study was tested using a PLS structural equation model.

## Data Analysis

Partial least squares were used to conduct the data analysis of this research in two steps. In the initial step of the analysis, the validity and reliability of the research model were analyzed. While, in the second step of data analysis, the path coefficients were analyzed. Construct’s validity and reliability, as well as their associations, were analyzed in the two steps of data analysis (Anderson and Gerbing, 1988; Hulland, 1999). PLS is one of the most popular research tools employed to analyze and manage the constructs’ relationships (Petter et al., 2007). Moreover, PLS is believed to be the best in managing the irregular distribution of constructs due to its user-friendly measures to calculate the normality and randomness of constructs. Furthermore, PLS can also calculate dynamic prediction frameworks (Chin and Newsted, 1999). Consequently, PLS was considered to be the suitable option to conduct the data analysis of this research.

### Outer Model and Validation

The internal reliability and validity of every construct were calculated in the analysis of the external model. The discriminant and convergent validity were also calculated for each item. The factor loading associated with each item was used to assess the reliability of every item. The threshold factor loading value of 0.6 was used to determine the reliability of each item (Hair et al., 2010). Table 1 shows the composite reliability (CR) of every construct. The CR was found to be higher than the threshold CR value of 0.7 for each construct in this study; hence, indicating that each construct was internally significant (Chin, 1998).

**TABLE 1:** Convergent validity.

Constructs	Items	Factor loadings	Cronbach’s alpha	Composite reliability	Average variance extracted	Adjusted *R*-square
Perceived enjoyment	PENJ1	0.779	0.659	0.811	0.590	
	PENJ2	0.813				
	PENJ3	0.708				
Perceived vividness	PVIV1	0.716	0.765	0.866	0.685	
	PVIV2	0.872				
	PVIV3	0.885				
Situational involvement	SINV1	0.832	0.722	0.840	0.637	
	SINV2	0.797				
	SINV3	0.764				
Challenge	CHAL1	0.854	0.844	0.906	0.763	
	CHAL2	0.846				
	CHAL3	0.917				
Confirmation	CONF1	0.836	0.695	0.828	0.620	
	CONF2	0.880				
	CONF3	0.624				
Flow experience	FLOW1	0.770	0.791	0.863	0.613	0.492
	FLOW2	0.792				
	FLOW3	0.776				
	FLOW4	0.791				
Perceived usefulness	PUSE1	0.774	0.734	0.849	0.653	0.207
	PUSE2	0.829				
	PUSE3	0.820				
Satisfaction	SATI1	0.883	0.874	0.923	0.800	0.488
	SATI2	0.930				
	SATI3	0.868				
Continuance intention	CINT1	0.851	0.836	0.901	0.752	0.427
	CINT2	0.883				
	CINT3	0.868				

The average variance calculated for each construct was also considered to measure the convergent validity. The values were analyzed with the threshold (AVE) value of 0.5. It was found that all the constructs possessed an (AVE) value higher than the threshold value of 0.5, hence, suggesting good convergent validity (Fornell and Larcker, 1981). Table 1 uncovers that the AVEs for the constructs measured in this study’s evaluation are between 0.692 and 0.824, implying significant convergence.

To evaluate the differences among the different constructs and research items, the discriminant validity was analyzed. Table 2 suggests decent discriminant validity for all the indicators measuring the constructs. The factor loading value of every indicator measuring a specific construct exceeds the factor loading value of all the other constructs in the latent structure (Hair et al., 2016).

**TABLE 2:** Standardized factor loadings and cross-loadings of the outer model.

	Perceived enjoyment	Perceived vividness	Situational involvement	Challenge	Confirmation	Flow experience	Perceived usefulness	Satisfaction	Continuance intention
PENJ1	0.779	0.417	0.266	0.368	0.406	0.373	0.353	0.416	0.410
PENJ2	0.813	0.536	0.267	0.208	0.291	0.351	0.433	0.318	0.450
PENJ3	0.708	0.602	0.105	0.041	0.271	0.245	0.521	0.224	0.449
PVIV1	0.470	0.716	0.251	0.276	0.351	0.317	0.269	0.383	0.421
PVIV2	0.574	0.872	0.260	0.209	0.297	0.292	0.521	0.341	0.547
PVIV3	0.576	0.885	0.283	0.285	0.382	0.369	0.565	0.382	0.601
SINV1	0.237	0.292	0.832	0.539	0.410	0.427	0.261	0.413	0.290
SINV2	0.203	0.286	0.797	0.549	0.397	0.378	0.205	0.403	0.274
SINV3	0.249	0.206	0.764	0.444	0.270	0.549	0.229	0.305	0.274
CHAL1	0.255	0.290	0.478	0.854	0.442	0.573	0.324	0.541	0.291
CHAL2	0.307	0.306	0.592	0.846	0.409	0.491	0.264	0.390	0.239
CHAL3	0.211	0.227	0.597	0.917	0.413	0.530	0.205	0.475	0.278
CONF1	0.391	0.326	0.335	0.493	0.836	0.382	0.342	0.611	0.434
CONF2	0.328	0.375	0.439	0.415	0.880	0.367	0.341	0.564	0.454
CONF3	0.288	0.295	0.245	0.159	0.624	0.220	0.279	0.276	0.405
FLOW1	0.347	0.407	0.362	0.356	0.264	0.770	0.309	0.332	0.404
FLOW2	0.257	0.269	0.409	0.373	0.347	0.792	0.238	0.356	0.329
FLOW3	0.405	0.281	0.433	0.477	0.331	0.776	0.334	0.399	0.397
FLOW4	0.327	0.294	0.589	0.661	0.372	0.791	0.277	0.459	0.269
PUSE1	0.448	0.379	0.220	0.160	0.308	0.308	0.774	0.250	0.408
PUSE2	0.449	0.424	0.239	0.335	0.389	0.313	0.829	0.341	0.340
PUSE3	0.435	0.530	0.248	0.238	0.291	0.281	0.820	0.386	0.440
SATI1	0.421	0.444	0.416	0.390	0.530	0.446	0.438	0.883	0.555
SATI2	0.379	0.437	0.401	0.514	0.586	0.454	0.355	0.930	0.528
SATI3	0.349	0.315	0.420	0.554	0.615	0.440	0.291	0.868	0.463
CINT1	0.500	0.610	0.247	0.183	0.415	0.341	0.448	0.413	0.851
CINT2	0.522	0.535	0.369	0.391	0.548	0.402	0.427	0.584	0.883
CINT3	0.437	0.520	0.289	0.215	0.431	0.405	0.402	0.492	0.868

*The factor loading value of every indicator measuring a specific construct exceeds the factor loading value of all the other constructs in the latent structure, The highest value of every indicator has been highlighted in yellow.*

### Inner Model Result and Hypotheses Testing

**FIGURE 2:**
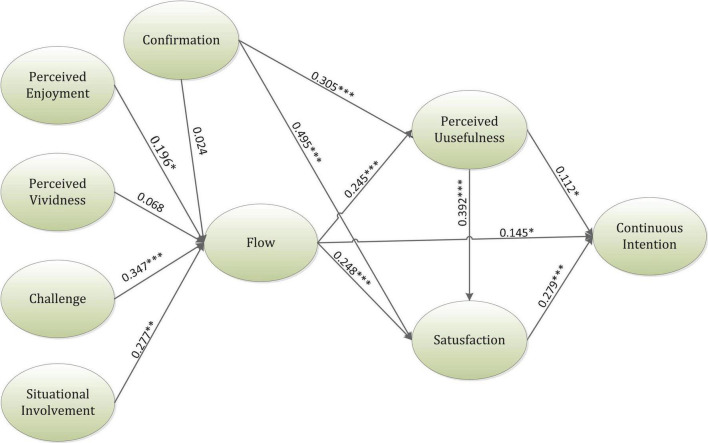
A framework of the inner model result. *p ≤ 0.05, **p ≤ 0.01, ***p ≤ 0.001.

An internal PLS model was employed to analyze the proposed relationships of this research. The values of path coefficients determined the strength and direction of every measured construct. Furthermore, the predictor variables’ percentage was signified by the value of *R*-square that demonstrated the predictive capability of the research framework. Bootstrapping was used to examine the degree of every path coefficient. Data resampling was used to re-extract the data, which was more accurate than the normal estimated value (Purvis et al., 2001). This research, hence, utilized this methodology to verify the relationship between constructs. Table 3 and Figure 2 reveal the inner model results of the hypotheses. According to the results indicated by Table 3, all the hypotheses, except Hypotheses 2 and 4, were accepted. The variables in Hypothesis 2 (β = 0.024, *T*-value = 0.369) and Hypothesis 4 (β = 0.068, *T*-value = 0.856) were not in a significant relationship. The online students’ flow experience was found to be in a significant relationship with continuous intention (β = 0.145, *T*-value = 2.475). The antecedents comprising perceived enjoyment (β = 0.196, *T*-value = 2.462), challenge (β = 0.347, *T*-value = 5.090), and situational involvement (β = 0.277, *T*-value = 4.311) were found to be in a positive relationship with flow experience; however, confirmation and perceived vividness did not have significant effects on the flow. Furthermore, flow and confirmation were found to be in a significant relationship with PU (β = 0.245, *T*-value = 3.573), (β = 0.305, *T*-value = 4.161) and satisfaction (β = 0.248, *T*-value = 4.189), (β = 0.495, *T*-value = 9.214). Moreover, PU was found to be in a significant relationship with satisfaction (β = 0.112, *T*-value = 2.251) and continuous intention (β = 0.279, *T*-value = 5.595). Finally, satisfaction was found to be in a significant relationship with continuous intention (β = 0.392, *T*-value = 6.771). This research study also analyzed some of the indirect effects, which are shown in Table 4.

**TABLE 3:** Hypotheses results.

Hypotheses	Path coefficient	*T* values	*P* values	Results
H1: Flow experience → continuance intention	0.145	2.475	0.013	Supported
H2: Confirmation → flow experience	0.024	0.369	0.712	Not supported
H3: Perceived enjoyment → flow experience	0.196	2.462	0.014	Supported
H4: Perceived vividness → flow experience	0.068	0.856	0.392	Not supported
H5: Challenge → flow experience	0.347	5.090	0.000	Supported
H6: Situational involvement → flow experience	0.277	4.311	0.000	Supported
H7: Flow experience → perceived usefulness	0.245	3.573	0.000	Supported
H8: Flow experience → satisfaction	0.248	4.189	0.000	Supported
H8: Confirmation → perceived usefulness	0.305	4.161	0.000	Supported
H9: Confirmation → satisfaction	0.495	9.214	0.000	Supported
H10: Perceived usefulness → satisfaction	0.112	2.251	0.024	Supported
H11: Perceived usefulness → continuance intention	0.279	5.595	0.000	Supported
H12: Satisfaction → continuance intention	0.392	6.771	0.000	Supported

**TABLE 4:** Indirect effects.

Hypotheses	Path coefficient	*T* values	*P* values	Results
Perceived enjoyment → flow experience → perceived usefulness → continuance intention	0.013	1.861	0.063	Not supported
Confirmation → satisfaction → continuance intention	0.194	5.117	0.000	Supported
Perceived vividness → flow experience → perceived usefulness → continuance intention	0.005	0.745	0.456	Not supported
Confirmation → perceived usefulness → satisfaction → continuance intention	0.014	1.761	0.078	Not supported
Confirmation → perceived usefulness → continuance intention	0.086	3.125	0.002	Supported
Confirmation → flow experience → perceived usefulness → continuance intention	0.002	0.346	0.729	Not supported
Flow experience → perceived usefulness → satisfaction → continuance intention	0.010	1.979	0.048	Supported
Situational involvement → flow experience → perceived usefulness → satisfaction → continuance intention	0.003	1.790	0.073	Not supported
Perceived enjoyment → flow experience → perceived usefulness → satisfaction → continuance intention	0.040	2.214	0.027	Supported
Challenge → flow experience → perceived usefulness → satisfaction → continuance intention	0.004	1.858	0.063	Not supported

## Discussion

Built on the integrated theoretical framework of antecedents of flow and ECM, this research investigates the way flow experience drives the online students’ intention to engage in online English teaching platforms. Thus, the findings are noteworthy. According to the findings of the study, the flow was found to be in a significant relationship with continuous intention. The outcomes of this study were found to be in accordance with Yung Ming Chen’s (Cheng, 2014) study. This study proposed a model founded on the flow theory, ECM, and DeLone and McLean’s (IS) framework to investigate if quality considerations impacted the nurses’ continuous intention to use the online learning platform. According to the outcomes of this research system, support service, instructor, and information quality were in a significant relationship with PU, confirmation, and flow that, in turn, impacts the nurses’ satisfaction and continuous intention of the online learning platform’s use. Furthermore, all the antecedents of flow, except confirmation and perceived vividness, were found to be in a significant relationship with the flow. The findings of the significant antecedents were found to be somewhat like other studies (Havitz and Mannell, 2005; Wu et al., 2021). However, regarding the relationships of challenge and flow, a small number of studies have described the associations of challenges and skills with flow experience regarding the objective of achieving an equilibrium of completing an activity (Wu et al., 2016).

The outcomes of this research suggested significant associations between the variables of flow and ECM. Flow state or ECM has been commonly considered for its impact on overall online platforms, however not much on specific online English learning platforms. The findings of this research were somewhat similar to the findings reported by other research studies, indicating that the ECM related to the technological side or flow experience is usually deemed as an essential interest for common online platforms (Floh and Madlberger, 2013; Tsao, 2013; Hsu and Lin, 2015). Specifically, the associations for ECM-related variables, for instance, confirmation with both PU and satisfaction, were fairly described in prior pieces of research related to online platforms and continuous intention (Hsu and Lin, 2015; Lu et al., 2019; Wu et al., 2020).

## Theoretical Implications

This study theoretically contributed an innovative research framework built on the flow antecedents, flow theory, and ECM. This research improves the knowledge about the impacts of flow antecedents to students’ perceptions of their satisfaction and continuous intention regarding usage of online English learning platforms and, therefore, delivers educational organizations wish to effectively facilitate students’ continued online learning platform use intention with implications and relevant recommendations. This study proposes three key issues: antecedents of flow, flow theory, and ECM, for defining online English learning underlying a students’ collaboration with online platforms. This model is established on a well-defined online English platform involving three phases. The first phase is for an assessment of flow with the continuous intention for learning on online platforms. The next is the impact of flow antecedents on flow experience in using online platforms. Finally, the evaluation of relationships between confirmation, PU, satisfaction, and continuous intention for online English platforms was measured by a modified ECM model. This research offers a new approach for flow experience in online platforms.

Moreover, although, during the COVID-19 pandemic, the online students’ segment signifies a rapidly growing segment, a small number of research studies have been conducted to target this segment in the wake of a theoretical framework planned for determining the continuous usage intention of online English learning platforms during the pandemic. This study has numerous contributions. For instance, this study contributed theoretically to flow by analyzing and determining variations related to the flow antecedents. Furthermore, this study related flow antecedents with the flow and continuous usage intention, hence, contributing to the flow theory in the online English learning students’ perspective. The findings of this study indicate that flow can enhance positive evaluations of PU and satisfaction. It emphasizes that experiencing flow, while using online English learning platforms will remove the irrelevant stimuli, making online English learning useful and satisfactory.

## Managerial Implications

There are numerous crucial practical implications. The dyadic nature of online English learning is characterized especially in this research, demonstrating the significance of both flow state issues and ECM for efficiently improving the motivation of online learning conduct. Although online learning is a student’s conduct for an emotive reaction to motivation by selected features or design in an online learning platform, the results would offer experts a guideline to construct an online learning platform to be efficient and desirable to their students. To improve students’ insight into the usefulness of online platforms for an improved online learning experience, managers can project for an extraordinary online learning platform containing several system characteristics, for instance, helpful information provided for students, an attractive online learning platform interface, a significant navigation instrument, and a rapid feedback mechanism. From an emotive viewpoint, a learning activity may directly connect to the perceived vividness and a content layout of an online platform that can be a huge challenge for online students. The content layout concerning task challenges can link to a huge number of online learning mechanisms to help corresponding continuous needs in order to increase the motivation of online learning. Furthermore, linking online learning challenges involves the obligations of skills, for instance, curriculum designs, and computer self-efficiency, once these abilities can be backed by the content layout of online English learning platforms. These abilities could significantly improve the continuous intention of online learning activities regardless of increased learning challenges. Finally, the questions proposed in this research with their empirical findings offer profound understanding of establishing a well-devised online English learning platform that can motivate online learning. These results and managerial implications for online English language platforms are innovative and significant in practice.

Moreover, flow can be considered as a foundation for the positive viewpoints of the internet and technology regarding online English learning platforms during the COVID-19 pandemic. Additionally, PU and satisfaction have vital roles to enhance the users’ experience of online English learning platforms. Corresponding to these findings, marketers have a firm belief that merging technological expertise with the flow can produce satisfactory experiences for online English learners. In contrast to other conventional learning methods, online learning platforms provide safety, reduce efforts, and enhance the learning experiences of the platform users during the pandemic. Moreover, practitioners are recommended to take into account the flow methodology while devising the marketing policies for every stage of the learning process for the online learning platform users. User frustration experienced during the learning process might cause the disappearance of the flow experience (Herrando et al., 2018). Consequently, to lessen the unfavorable effects of other stages of the online learning process, online learning platforms are required to employ strategies to keep the user up-to-date concerning the probability of flow.

## Limitations and Future Research

Future investigations, in general, can incorporate other viewpoints, including culture and media communication to comprehend online English learning behavior. Especially, since this study deals with improvement issues of online platforms, numerous technology-use frameworks can be studied as substitutes to discover various key interests related to online learning behavior, like the IS success model, the uses and gratifications theory (Petter et al., 2013), the theory of planned behavior (Ajzen, 2011), and their modifications. A flow state can be expanded by a hedonistic framework, including external and internal motivation. Second, it can be claimed that flow can be regarded as a multidimensional construct, while it is depicted as a single-dimensional construct in this research. Hence, there is a shortage of comprehensive knowledge of flow and its correlation with online English platforms’ PU, satisfaction, and continuous intention. Upcoming research is supported to incorporate multidimensional flow constructs and analyze them. Finally, the research framework of this study is based on flow antecedents and theory, and the ECM to describe students’ online learning intention. It is considered that more theoretical models, like self-determination theory, could be incorporated into future studies to enhance their utility in describing students’ online learning activities. This research incorporated a number of flow antecedents to measure the online learning experience, which can produce generalized outcomes. Hence, future researchers are advised to select specific constructs to get more focused viewpoints and outcomes. Furthermore, this study signifies the role of PU in the online English learning platforms for students during the pandemic. According to previous studies, the use of the internet and technology was not considered to be important by many users in their daily lives, and, hence, future studies can assist online English learning students to acknowledge the importance and benefits of the internet and online learning platforms during the pandemic (Frissen, 2005; Barta et al., 2021). In addition, future studies can target designing a user-friendly interface for online learning students and integrate the essential features, such as bright colors, large fonts, clear video, and audio for online learning platforms. The user-friendly platform can reduce stress levels and additionally generate a flow experience in online learning students.

## Data Availability Statement

The raw data supporting the conclusions of this article will be made available by the authors, without undue reservation.

## Ethics Statement

Ethical review and approval were not required for the study on human participants in accordance with the local legislation and institutional requirements. Informed consent was obtained from all the subjects involved in the study.

## Author Contributions

HZ and AK: conceptualization, formal analysis, methodology, validation, and writing – the original draft, reviewing, and editing. HZ: investigation. All authors have read and agreed to the published version of the manuscript.

## Conflict of Interest

The authors declare that the research was conducted in the absence of any commercial or financial relationships that could be construed as a potential conflict of interest.

## Publisher’s Note

All claims expressed in this article are solely those of the authors and do not necessarily represent those of their affiliated organizations, or those of the publisher, the editors and the reviewers. Any product that may be evaluated in this article, or claim that may be made by its manufacturer, is not guaranteed or endorsed by the publisher.
